# Cost analysis of a coaching intervention to increase use of transradial percutaneous coronary intervention

**DOI:** 10.1186/s43058-021-00219-5

**Published:** 2021-10-27

**Authors:** Kevin I. Duan, Christian D. Helfrich, Sunil V. Rao, Emily L. Neely, Christine A. Sulc, Diana Naranjo, Edwin S. Wong

**Affiliations:** 1 Health Services Research and Development, VA Puget Sound Health Care System, Seattle, WA USA; 2 Division of Pulmonary, Critical Care, and Sleep Medicine, University of Washington, 1959 Northeast Pacific Street, Box 356522, Seattle, WA 98195 USA; 3 Department of Health Systems and Population Health, University of Washington, Seattle, WA USA; 4 Durham VA Health Care System, Durham, NC USA; 5 Duke Clinical Research Institute, Durham, NC USA

## Abstract

**Background:**

The transradial approach (TRA) to cardiac catheterization is safer than the traditional transfemoral approach (TFA), with similar clinical effectiveness. However, adoption of TRA remains low, representing less than 50% of catheterization procedures in 2015. Peer coaching is one approach to facilitate implementation; however, the costs of this strategy for cardiac procedures such as TRA are unclear.

**Methods:**

We conducted an activity-based costing analysis (ABC) of a multi-center, hybrid type III implementation trial of a coaching intervention designed to increase the use of TRA. We identified the key activities of the intervention and determined the personnel, resources, and time needed to complete each activity. The personnel cost per hour and the activity duration were then used to estimate the cost of each activity and the total variable cost of the implementation. Fixed costs related to designing and running the implementation were calculated separately. All costs are reported in 2019 constant US dollars.

**Results:**

The total cost of the coaching intervention implementation was $374,863. Of the total cost, $367,752 were variable costs due to travel, preparatory work, in-person coaching, post-intervention evaluation, and administrative time. We estimated fixed costs of $7112. The mean marginal cost of implementing the intervention at only one additional medical center was $52,536.

**Conclusions:**

We provide granular cost estimates of a conceptually rooted implementation strategy designed to increase the uptake of TRA for cardiac catheterization. We estimate that implementation costs stemming from the coaching approach would be offset after the conversion of approximately 409 to 1363 catheterizations from TFA to TRA. Our estimates provide benchmarks of the expected costs of implementing evidence-based, but expertise-intensive, cardiac procedures.

**Trial registration:**

ISRCTN, ISRCTN66341299. Registered 7 July 2020—retrospectively registered

**Supplementary Information:**

The online version contains supplementary material available at 10.1186/s43058-021-00219-5.

Contributions to the literature

- Economic evaluation remains limited in implementation science. Few studies provide detailed cost analysis as part of implementation trials, particularly related to coaching interventions for medical procedures.
- We used an activity-based costing methodology to provide a detailed cost analysis of an implementation trial aimed at increasing the use of the transradial cardiac catheterization technique.
- Our cost estimates can be used by health system leaders seeking to expand the use of transradial cardiac catheterization in their facilities. Our analysis and methodology serve as a model to other implementation trials planning to perform cost analysis.

## Background

Cardiac catheterization is a common cardiac procedure used in the treatment of coronary heart disease [1]. Catheterization can be performed by accessing either the femoral artery in the groin or the radial artery in the wrist [2, 3]. The transfemoral approach (TFA) has historically been the most common method of performing cardiac catheterization in the United States (US) [4]. However, subsequent research demonstrates that the transradial approach (TRA) is preferred, due to improved outcomes, decreased complications, and lower costs [5–8]. This evidence supporting TRA is reflected in professional society clinical practice guidelines, which have been updated to state a preference for TRA [9, 10]. In the US, implementation of TRA has been improving (25% in 2014), but still lags behind TRA rates in Europe and Asia (e.g., almost 50% in the UK in 2012) [5, 11, 12].

Within the US Department of Veterans Affairs (VA), TRA accounted for 36% of diagnostic catheterizations and 32% of percutaneous coronary interventions (PCI) by 2015 [13]. The predominance of the femoral approach persists despite up to 94% of VA cardiologists rating TRA as superior to TFA on almost all counts: safety, patient comfort, and ease of monitoring patients post-procedure [14]. Furthermore, use of TRA in VA is heterogeneous, with several VA catheterization labs accounting for the majority of VA-wide TRA volume [15].

Several barriers have prevented broader uptake of TRA both within and outside of VA [14, 16, 17]. For cardiologists inexperienced in TRA, the approach initially takes significantly longer, incurs higher radiation exposure due to increased fluoroscopy use, and results in more crossovers (when an operator begins a procedure via one route but has to complete it via the alternative route) [18–20]. Because TFA effectively achieves the same end as TRA, it is costly for cardiologists who are already proficient in TFA to invest sufficient time and effort to master new skills to execute TRA. Moreover, the TRA learning curve is not linear, with operators exhibiting slow improvement initially, so they likely perceive little payoff from their initial efforts to perform TRA [21]. Similar costs in time and effort exist for other members of the cardiac catherization team: case preparation, equipment selection, and post-procedure monitoring all change in small but important ways for TRA and take time to become routine [22].

Every complex skill presents a learning curve, and coaching by an experienced peer has long been demonstrated as a method of shortening the learning curve and improving skill acquisition in medicine [23]. Peer coaching helps inexperienced clinicians develop new heuristics (i.e., mental shortcuts for performing the skill) and routines (i.e., sets of inter-dependent actions among team members) [24]. This is achieved through demonstration of skills, explanation of underlying thinking and approaches, and corrective feedback [24]. However, the challenge of peer coaching is that it is time intensive and requires access to an experienced peer, who may be difficult for operators to identify and geographically remote. We sought to test a peer-coaching intervention to increase the speed uptake of TRA. Clinical trial ISRCTN66341299 was a stepped-wedge implementation trial of a coaching intervention designed to increase uptake of TRA at 7 Veterans Affairs Medical Centers (VAMCs) in the US. The coaching strategy was informed by (1) conceptual models related to implementation and the theory of expert skill development [25, 26] and (2) prior empirical findings related to how clinical practices adopt new skills [22, 27–29].

Cost and financial resources are often cited as a barrier to the adoption of evidence-based practices (EBPs) such as TRA [30]. As such, there is a need to better describe costs in implementation trials to provide health system leaders a full understanding of the economic ramifications of implementation. Transparent and full cost analysis is also an important step towards the reproducibility of strategies to improve uptake of EBPs. While there is an increasing inclusion of cost analyses in clinical drug trials, there are few cost analyses in implementation trials [31, 32]. The goal of the present analysis was to examine the cost of the coaching implementation strategy from the implementation trial.

## Methods

### Study setting and implementation trial overview

**Fig. 1:**
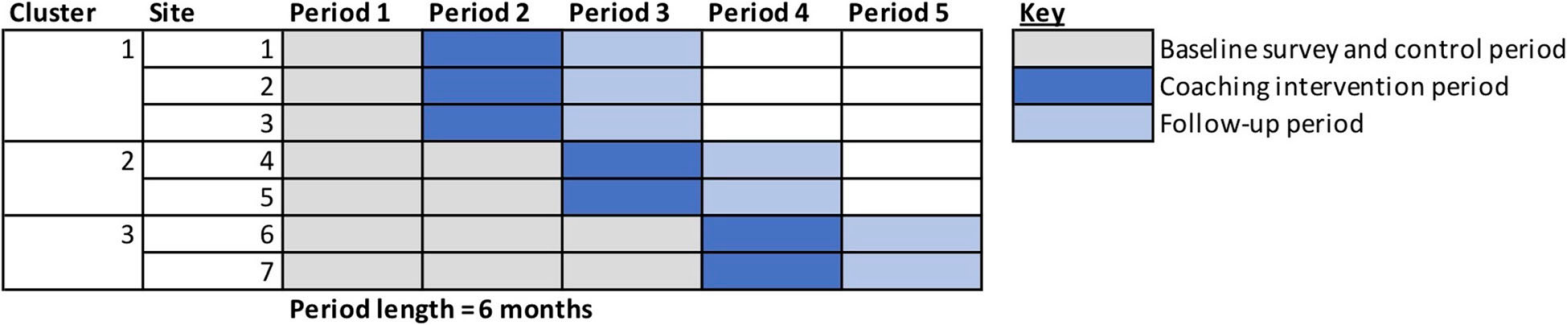
Stepped wedge design

This descriptive economic analysis examined implementation costs within a hybrid type-III implementation trial (Clinical Trials Identifier: ISRCTN66341299). The trial took place from March 2018 to March 2020. A stepped-wedge trial design was employed such that catherization teams at seven VAMCs were randomized (in sets of two to three sites) to receive a coaching intervention during one of three steps, each four months apart (Fig. 1). Cardiology-specific site characteristics are summarized in Table 1. The trial compared the rate of TRA use before and after the coaching intervention. The stepped-wedge design allowed sites to serve both as internal controls in pre-post comparison and to control for secular trends by comparing sites between steps. The control arm was usual care pre-intervention. At the end of the trial period, catherization teams at all sites had received the coaching intervention. Each site was represented by a catherization lab team, comprising a cardiologist with 1–2 nurses and/or radiology technicians (i.e., 2–3 members per catherization lab team). The coaching intervention (described in detail in the “Coaching implementation strategy” section) was designed to mitigate a well-documented learning curve for TRA [18, 19]. The underlying hypothesis was that most operators, trained on TFA, never perform TRA consistently enough to achieve proficiency. On the rare occasions they do perform TRA, they tend to perform it on the lowest risk patients who benefit the least [33]. The coaching intervention was designed to reduce the learning curve and introduce external accountability. The primary outcome was procedure-level odds of TRA, and secondary outcomes were clinical outcomes expected to improve with TRA, such as bleeding complications, and measures of TRA proficiency, including contrast volume and an implementation fidelity checklist completed at the conclusion of the coaching visit.

**Table 1:** Site characteristics

	Participants (***n*** = 7 sites)	Non-participants (***n*** = 73 sites)*
**Interventional cardiologists (mean, SD)**	4.7 (0.8)	3.7 (1.9)
**Diagnostic case volume (mean, SD)**	595 (226)	410 (219)
**% TRA (mean, range)**	27% (2.5–64%)	54% (0–93%)
**PCI volume (mean, SD)**	230 (139)	126 (934)
**% TRA (mean, range)**	22% (2–44%)	46% (0–95%)
**Facility bed size (mean, SD)**	135 (66)	103 (42)
**Academic affiliation (*n*, %)**	7 (100%)	73 (100%)
**Onsite surgical backup (*n*, %)**	4 (57%)	37 (51%)

*Abbreviations*: SD, standard deviation; TRA, transradial approach; PCI, percutaneous coronary intervention

*Non-participant sites include all VA facilities that have a cardiac catheterization lab

### Coaching implementation strategy

The coaching intervention included two primary components: (1) an in-person team-based training at an experienced catheterization lab, and (2) a participant site visit by the coaching team approximately 1–2 months later, during which coaches observed a series of pre-scheduled TRA cases performed by the participants, and provided feedback and support. The training component consisted of a day-long training course that included three elements: (1) didactic sessions, (2) simulation, and (3) observing cases performed by TRA coaches (see Additional file 1 for course agenda). Catheterization teams sent 2 to 3 participants (one cardiologist along with a cardiac catheterization nurse and/or radiology technician) to the training course held at a central site, which was capable of hosting up to 10 catheterization lab teams at a time. The course began with didactic lectures for all participants followed by research on TRA, information on common issues encountered and discussion of the TRA technique. In the afternoon, the participants observed live cases performed by a highly proficient team. Participants also observed pre-procedure set-up, and post-procedure care and monitoring. The cases ranged from diagnostic to PCI cases.

Approximately 1 month following participation in the training course, an experienced interventionalist and nurse (members of the coaching team) conducted a 1-day coaching visit to the participants’ catheterization lab. The purpose of this visit was to address any challenges, including helping teams recognize opportunities for improvement. Coaching visits involved coach/participant meetings and observing live cases. The coaching visit also involved supervised, hands-on training. A coaching approach was important because it focused on corrective, non-punitive feedback. The coaching visit was timed to occur after the participants had returned to their catheterization lab and had an opportunity to apply what they learned at the training course and encounter challenges. At the time of the coaching visit, the participant sites were likely not yet proficient and therefore at risk of not adopting TRA. It is at this high-risk point that we believed coaches could help troubleshoot and offer encouragement.

### Data sources

The primary source of data were detailed study records tracking attendance and time involved in meetings and seminars related to the coaching strategy. Costs associated with travel were derived from VA travel reimbursement records. Interview logs were used to ascertain the time devoted to coaching interviews. We surveyed a random sample of implementation participants to ascertain time devoted to preparation for implementation activities. Salaries of participants used to monetize time devoted to the coaching strategy were derived from VA Personnel and Accounting Integrated Data (PAID).

### Costing methodology overview

Implementation costs for the coaching strategy primarily consist of the opportunity cost of time devoted to implementation activities [34]. To calculate these costs, we conducted activity-based costing (ABC). ABC is a method of calculating cost that was first developed in the business sector and has since been applied to health care settings [35]. As implied by the name, this method of micro-costing relies on dividing a service or product—in our case, the coaching intervention—into sub-component activities. The cost of each activity can then be estimated by calculating both the labor cost per unit time of involved personnel and the length of time it takes to complete a given activity to estimate the total cost. This technique, and its derivative method, time-driven activity-based costing (TDABC), have been previously used in cost studies in health care, but there has been limited use in implementation science, particularly in implementation trials [36, 37]. We chose to use traditional ABC, and not TDABC, given the time inputs available from participants for our analysis. For this study, we took the perspective of the health care system in calculating cost. We excluded personnel time and activities that were directly related to research, such that the results of this study are representative of implementation of the coaching intervention alone. Research staff that attended the training sessions were excluded from the cost analysis. Administrative time devoted to research was excluded by staff estimates of percent full-time equivalents (FTE). As an overview of our ABC methodology, we followed the following steps: (1) we divided the coaching intervention into sub-component activities that were then categorized as variable costs (travel, preparatory work, in-person coaching, post-intervention evaluation and administrative time) and fixed costs (intervention development, computers). (2) We determined labor costs per hour for each participant and coach. (3) We collected time estimates for each activity and personnel type from surveys, activity logs, conference agendas, and key informants. (4) We obtained full study costs for each activity by multiplying personnel-level time estimates (in hours) with the individual hourly wage rate and summed across all three randomization clusters. We then estimated per VAMC and per cluster average costs by repeating the calculation for the full study cost described above, but only including participant personnel assigned to a VAMC or cluster. For the per VAMC and per cluster calculations, coach costs were divided and equally attributed across VAMCs and clusters. Finally, we estimated costs per personnel, per cluster by multiplying average labor cost for each personnel type with the average time spent on each activity by each personnel type.

### Labor cost per hour

A key input variable in calculating variable and fixed costs using the ABC method is the labor cost per unit time. We calculated the labor cost per hour for a given individual by dividing their total annual salary by the number of working hours per year. Total annual salary was inclusive of bonus, performance pay, and a 30% increase to reflect the cost of benefits [38]. To calculate the working hours per year, we assumed 261 weekdays per year, 8-h workdays and an “applied rate” of 80% for all participants. The applied rate is defined as the proportion of an employee’s time that is devoted to productive working hours (i.e., excluding vacation, holidays, sick leave and continuing medical education). The VA Health Economics Resource Center recommends using a rate of 80–85% for VA salaries, and we selected the lower bound of the recommended range for a conservative estimate [38]. We then used the calculated labor cost per hour for a given individual to calculate the variable and fixed costs, by multiplying the number of hours an individual spent on a given activity by the labor cost per hour.

### Variable costs

We subcategorized the variable costs into five components: (1) travel, (2) preparatory work, (3) in-person coaching, (4) post-intervention evaluation, and (5) the administrative time devoted to logistical support and coordination of the implementation. These costs are categorized as variable costs, because they varied with each randomization cluster based on the number of personnel and the amount of travel that was involved in a given cluster. For the components of travel, preparatory work, in-person coaching, and post-intervention evaluation, the time spent performing each activity was multiplied by the labor cost per hour for a given individual to estimate the cost of the activity. Travel primarily consisted of trips to the 1-day learning lab and coaching visits by the TRA coaching team to each participating site. Direct travel costs related to flights, hotels, and other travel-related expenses were obtained from travel reimbursement claims and the VA travel software system. The time spent in transit for personnel that traveled was estimated using the Google Flights website [39]. Local personnel incurred no travel costs. The administrative time spent coordinating travel was based on the percent FTE devoted to this task estimated by project administrative staff. The preparatory work primarily entailed the coaches updating presentation materials for the didactic portion of the learning lab, coordinating the learning lab schedule with the catheterization laboratory’s clinical schedule, and meetings before and after the learning lab and coaching visit to huddle and debrief. For preparatory work, we obtained estimates from key informants of the average time coaches and participants spent reviewing materials prior to the in-person learning lab and in-person coaching visits. In-person coaching was described above in the “Coaching implementation strategy” section. For in-person coaching time, we obtained meeting agendas for both learning labs and coaching visits to estimate the time spent in coaching sessions. Post-intervention qualitative evaluation entailed telephone interviews (generally about 20 min) about participant experience and an online survey assessing their perceptions of TRA vs TFA, barriers to TRA, and workplace climate. Time spent by participants on post-intervention activities was estimated using the average of survey time logs and exit interview times. These average times were applied to all participants. Finally, the costs associated with administrative activities were obtained by estimating the percent FTE devoted to tasks required to coordinate and run the intervention by administrative staff. These duties include conducting the post-intervention evaluation interviews and surveys described above. The administrative time devoted to arranging travel was calculated separately as part of travel. The cost of materials was assumed to be minimal as teaching materials were transmitted electronically.

### Fixed costs

The main fixed costs were the time devoted to coaching intervention development and the cost of computing resources. Intervention development entailed establishing the initial schedule and content for the learning lab, drafting the didactic content, and developing an implementation fidelity checklist. The coaching intervention development was considered a fixed cost since the same intervention was applied to all participating sites. To estimate the one-time cost of developing these materials, we reviewed the in-person coaching agendas of both the classroom sessions and the site visits to ascertain the actual number of presentations delivered. Estimates of the time spent developing each presentation was estimated by the coaches. We included the cost of computing resources as a fixed cost, annuitized using a 5-year linear depreciation based on the recommended product lifespan of computers that is outlined by the United States Internal Revenue Service property depreciation recommendations [40]. We then attributed this annual cost to the 2 years during which the intervention was conducted. The cost of purchasing computer equipment was obtained by averaging the 10 most common products in a government price catalog [41].

### Analysis

We report cumulative implementation cost for the full study across the 7 participating sites for each cost subcomponent (e.g., fixed, variable costs). Next, we report the mean marginal cost of adding one VAMC to an existing coaching intervention program by calculating the average variable cost per VAMC. We also present univariate statistics of VAMC-level implementation costs including means, medians, standard deviations (SD) and interquartile ranges. Finally, we report the cost of implementing the intervention at only one VAMC (i.e., the startup cost of starting the coaching intervention de novo), which includes the entire fixed cost, as well as the mean variable costs for one VAMC.

### Sensitivity analyses

We performed multiple sensitivity analyses. First, we conducted a sensitivity analysis to evaluate how changes to travel inputs affected implementation costs. A significant amount of travel was involved for both coaches and participants for this nationwide implementation trial. A similar coaching intervention implemented at a regional level may yield lower costs if appropriate expertise is available. Therefore, in this sensitivity analysis, we assumed no air travel took place and that participant sites were located 1 h away from a hypothetical centralized center of TRA expertise. We also assumed no administrative time arranging travel. Second, we conducted a sensitivity analysis of how the same intervention would cost outside of the VA. VA salaries are generally lower than in community practice, which may underestimate total implementation costs if the coaching intervention was employed outside of VA [42]. Therefore, our second sensitivity analysis utilized national mean salaries for community interventional cardiologists and catheterization lab staff from 2019, instead of VA salaries [43, 44]. Furthermore, conference room space was not rented since all training was done in federal facilities at zero cost. Correspondingly, the second sensitivity analysis also included the cost of renting small conference rooms from federal facilities, estimated using the US General Services Administration list of meeting facilities [45]. Third, we conducted a sensitivity analysis of the cost of training all of the interventional cardiologists at the study sites. We applied the mean number of interventional cardiologists (Table 1) to all 7 study sites and used the site-specific mean salary of cardiologists in order to perform this sensitivity analysis.

Cost analysis was performed using Microsoft Excel. All costs were inflation adjusted to 2019 dollars using the Consumer Price Index [46, 47]. We adhered to the CHEERS reporting guidelines where appropriate.

## Results

### Time devoted to activities and labor cost per hour

Within a given randomization cluster, participants spent a mean of 4.5 h preparing ahead of organized coaching activities and a mean of 13.6 h at in-person sessions (training session and site visit). In total, physician participants devoted a mean of 24.7 h (SD = 2.2) to the intervention and nurse/radiology technicians devoted a mean of 24.0 h (SD = 2.4) to the intervention for a given randomization cluster (Table 2). Compared with participants, coaches spent more time traveling, but less time preparing for sessions ahead of organized activities and at in-person sessions. Labor costs per hour were similar between participants and coaches, within each type of role (physician and nurse/radiology technician, respectively).

**Table 2:** Personnel-level characteristics, mean time per activity, and costs per personnel per randomization cluster

	Participant—Physician (mean ***n*** = 2)	Participant—Nurse/Radiation Technician (mean ***n*** = 2.67)	Coach—Physician (mean ***n*** = 4.33)	Coach—Nurse/Radiation Technician (mean ***n*** = 1.67)
**Characteristics**				
Years in practice (median, IQR)	12 (9.25–17.75)	10.5 (2.5–20.5)	N/A****	N/A****
**Time per activity**				
Travel, (hours; mean (SD))	6.1 (2.1)	5.4 (2.4)	7.0 (6.8)	13.0 (4.8)
Preparation (hours; mean (SD))**	4.5***	4.5***	0.7 (0.3)	0.9 (0.2)
In-person coaching (hours; mean (SD))	13.6 (0.08)	13.6 (0.06)	9.5 (3.4)	12.0 (2.3)
Evaluation (hours; mean (SD))	0.5 (0.06)	0.5 (0.06)	0****	0****
Total time (hours; mean (SD))	24.7 (2.2)	24.0 (2.4)	17.2 (9.7)	25.9 (6.4)
**Costs**				
Variable costs				
Labor cost/hr (2019 dollars; mean (SD))	$269 (30)	$70 (23)	$257 (15)	$75 (4)
Total labor cost (2019 dollars; mean (SD))	$6652 (1028)	$1690 (574)	$4450 (2570)	$1952 (559)
Direct travel cost (2019 dollars; mean (SD))	$982 (281)	$1096 (181)	$1142 (737)	$1783 (738)
Apportioned administrative cost (2019 dollars; mean (SD))	$3683*****	$3683*****	N/A*****	N/A*****
Total variable cost (2019 dollars; mean (SD))	$11,318 (1079)	$6469 (695)	$5593 (3193)	$3735 (1285)
Apportioned fixed cost (2019 dollars; mean (SD))	$474*****	$474*****	N/A*****	N/A*****
**Total cost (2019 dollars; mean (SD))**	**$11,792 (1079)**	**$6943 (695)**	**$5593 (3193)**	**$3735 (1285)**

*Abbreviations*: SD, standard deviation; IQR, interquartile range

Mean time per activity and personnel-level costs are calculated as averages of the activities of a given coach/participant within a randomization cluster

**Learning time for participants, preparation time for coaches

***Based on reported estimates by key informants, applied to both physician and nurse/technologist roles, no SD reported

****Coaches did not participate in post-intervention evaluations or report years in practice

*****Fixed costs and administrative portion of variable costs were apportioned equally to participants only, as the target of the intervention. No SD reported

### Variable costs

The total variable cost was $367,752 across 7 VAMCs (Table 3). In terms of variable cost subcomponents, travel costs were $230,979 ($43,318 direct costs, $44,256 travel time cost, $143,405 travel-specific administrative time), preparatory costs were $12,583, in-person coaching costs were $67,959, post-intervention evaluation costs were $984, and general administrative time costs were $55,247. The mean marginal cost per VAMC was $52,536 (SD = $4530), and the median was $54,575 (IQR = $49,985–$55,391).

**Table 3:**
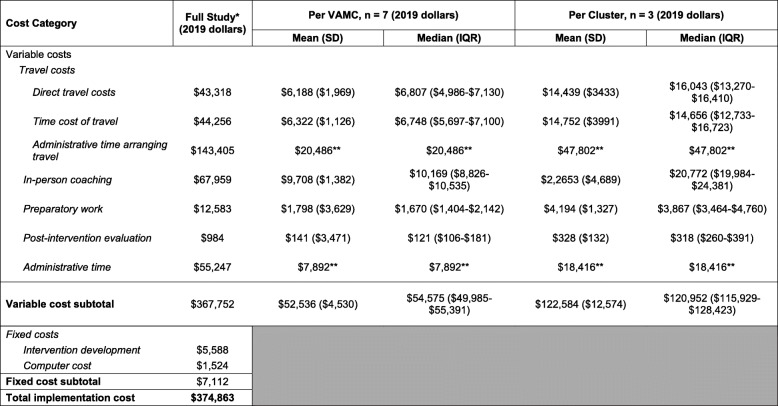
Variable, fixed, and total costs for the full study, per site and per randomization cluster

*Abbreviations*: VAMC, Veterans Affairs Medical Center; SD, standard deviation; IQR, interquartile range

*Means and SD were not calculated as this was total aggregate cost, and not average

**Administrative time was divided across 7 VAMCs or 3 clusters as an estimate. No SD or IQR is reported

### Fixed costs

The total fixed cost of the intervention was $7112 (Table 3), which consisted of intervention curriculum development ($5588) and computer cost ($1524).

### Total costs

The total overall cost of implementation of TRA coaching intervention was $374,863 for 7 VAMCs (Table 3). The mean marginal cost of adding one VAMC to the implementation program was $52,536 as described above. The total cost of implementing the TRA coaching intervention at one VAMC was $59,648, which was the sum of fixed costs ($7112) plus the mean marginal cost of adding one VAMC.

### Sensitivity analysis

When assuming 1 h of local travel with no air travel, accommodation or administrative time spent arranging travel, the total overall cost of implementation of the TRA coaching intervention was $156,796 for 7 VAMCs (Additional file 1). The mean marginal cost of adding one hospital to the implementation program using only local travel was $21,383. The total cost of implementing the TRA coaching intervention at one hospital was $28,495, which included $7112 in fixed costs plus the mean marginal cost from our sensitivity analysis. When assuming a non-VA setting of implementation by including national average salaries and room rental costs, the total overall cost of implementation of the TRA coaching intervention was $394,860 for 7 VAMCs (Additional file 1). When assuming that all interventional cardiologists at each study site were trained, the total overall cost of implementation of the TRA coaching intervention was $577,883 for 7 VAMCs (Additional file 1).

## Discussion

This study describes the costs of implementing a coaching intervention to increase use of TRA for cardiac catheterization within a large integrated health system using an ABC methodology. We estimated a total implementation cost of $374,863. Considering the per catheterization savings of $275–$916 (depending on the type of case) associated with TRA [7, 48, 49], we estimate that the implementation cost is approximately equal to converting 409–1363 cases from TFA to TRA. At the time of enrollment, VA sites eligible for our study performed an average of 650 catheterizations annually with a median TRA rate of 28%. As such, potential savings associated with implementing TRA catheterizations could be realized within only 1–2 years.

The majority of the implementation cost (88%) was driven by the high opportunity cost of personnel time. Specifically, time spent traveling to the in-person sessions, paying for travel expenses, and making travel arrangements represented 62% of the total implementation cost. Due to the administrative complexity of arranging travel through the VA, a significant amount of administrative effort was spent on this activity, which accounted for 38% of total costs.

Implementation costs could be dramatically reduced by conducting part, or all, of the coaching implementation strategy virtually. This would also facilitate access to TRA experts for health systems without internal experts. The didactic portion could be easily delivered remotely with current technology, either via remote teleconferencing or pre-recorded lectures. Emerging technologies such as virtual reality (VR) and haptic feedback devices could allow participants to experience the TRA-case portion of the coaching intervention remotely [50]. It may even be possible to create libraries of VR-recorded cases or simulated game cases representing common problems experienced during TRA catheterization. However, at this point, evidence is sparse about the relative effectiveness and costs of virtual versus in-person coaching [50]. Alternatively, a regional implementation strategy could not only reduce time spent traveling, but it would also reduce direct travel costs and the administrative burden. Our sensitivity analysis found that a regional strategy involving only driving locally and excluding the high administrative burden or coordinating travel reduced total implementation costs by 58% to a total of $156,795.91. However, a regional strategy may not be feasible if expertise is limited.

As health systems consider implementing interventions to increase uptake of the TRA, this analysis provides detailed cost estimates that can be used in budgetary analyses. Furthermore, this coaching intervention and its associated implementation costs serve as a useful model for broader dissemination of other interventional cardiology procedures. The use of minimally invasive cardiovascular procedures is growing [51] due to their favorable safety and efficacy profiles [52, 53]. Procedures like transcatheter aortic valve replacement (TAVR) currently remain concentrated in centers of expertise. However, with a recent change in the Centers for Medicare and Medicaid Services procedural volume requirements, many more hospitals will be eligible to perform these procedures [54]. As growth of new catheter-based cardiovascular procedures accelerates, coaching interventions like the one described in this study will likely be needed as access is expanded. This cost analysis and the methods used can serve as the basis of future implementation trials for other coaching interventions to foster uptake of evidence-based cardiology procedures.

There are several limitations to this study. First, the TRA coaching intervention was implemented within the VA, a large, integrated health care system in the US. As such, certain aspects of the reported cost estimates may not be directly generalizable to other settings. Specifically, VA travel costs are higher due to fare class requirements for airline tickets, and VA salaries are generally lower than those in the community [42]. At the same time, it is also possible that implementing a TRA intervention outside of an integrated network like the VA (i.e., across different health care systems) could prove to be more time-intensive and costly than our experience within the VA. We performed sensitivity analysis to enhance generalizability of cost estimates outside of the VA. Second, several estimates of the time required to complete a given activity (such as preparatory time and intervention development) were obtained by key informants. These estimates were subject to recall bias. Other estimates of activity time such as in-person coaching, and travel time are more accurate as they were based on meeting records and flight times. Third, we did not collect detailed data on how the duration and intensity of training varied across sites, which prevents analysis of potentially important patterns of site-level variation. However, given the structured nature of the intervention and the short time frame of the sessions (1 day each for the training program and site visit), large variation in time is unlikely. Similarly, we did not collect data regarding an individual’s baseline experience with TRA as a surrogate for how ready they were to transition to the TRA. Baseline experience may have an important role in contributing to variation in cost at the participant-level. Prior to the intervention, only 22–27% of cardiac catheterization cases at our study sites were performed using TRA (Table 1). This rate of TRA adoption would be considered low, given that TRA is now regarded as the default approach and should represent the majority of cases [9]. We expect settings with similarly low levels of baseline TRA experience would lead to similar costs to our study. In settings with higher levels of baseline TRA experience, we expect costs to be lower than reported in our study because the 1-day training course and 1-day site visit could be shortened or consolidated. Fourth, we excluded costs related to research from this analysis, so that our cost estimates represent implementation alone. However, it can be difficult for participants to distinguish between research and implementation in real-world settings, which could have led to over- or under-estimation of implementation costs [55]. Fifth, this cost analysis included the costs of activities that were part of pre-implementation planning and the implementation itself. Sustainment of the intervention after implementation was not part of the implementation trial. As a result, costs associated with these activities were not included in the estimates reported. Finally, given that only one cardiologist and 1–2 nurse/technicians participated from each site, our supposition was that other staff at participating sites would learn TRA through local observation and teaching. We made this supposition based on the many benefits to TRA and easily teachable techniques. However, if this does not occur in practice, our cost estimates will be an underestimate, as we estimated in our sensitivity analysis that formally training all cardiologists would be much more expensive.

## Conclusions

In conclusion, this study provides a detailed cost analysis of the implementation of a coaching intervention to increase usage of TRA in cardiac catheterization. The use of the ABC methodology provides granular and transparent cost estimations that enhance the reproducibility of the intervention and the ability of health system leaders to make informed budgetary decisions when considering broader adoption.

## Supplementary Information

**Additional file 1.**

**Additional file 2.** Completed CHEERS reporting guidelines.

## Data Availability

The data used for this study includes identifiable, private salary data and is therefore not available.

## Abbreviations

EBPs: Evidence-based practices
FTE: Full-time equivalents
PAID: VA Personnel and Accounting Integrated Data
PCI: Percutaneous coronary intervention
TAVR: Transcatheter aortic valve replacement
ABC: Activity-based costing
TFA: Transfemoral approach
TRA: Transradial approach
US: United States
VA: US Department of Veterans Affairs
VAMCs: Veterans Affairs Medical Centers
