# Supplementary material for: Cost analysis of a coaching intervention to increase use of transradial percutaneous coronary intervention
Source: Implement Sci Commun. 2021 Oct 27;2:123. doi: 10.1186/s43058-021-00219-5 (PMC8554885; doi:10.1186/s43058-021-00219-5)
Supplement: Supplementary file 1 — Additional file 1. [file 43058_2021_219_MOESM1_ESM.docx]

**Sensitivity Analysis #1: Local Travel Scenario**

Assumptions:

- No air travel or administrative time arranging travel
- Participants sites located 1 hour away from a hypothetical centralized center of TRA expertise

***Variable, Fixed and Total Costs For the Full Study, per Site and per Randomization Cluster***

| **Cost Category** | **Full Study***  **(2019 dollars)** | **Per VAMC, n = 7 (2019 dollars)** | | **Per Cluster, n = 3 (2019 dollars)** | |
| --- | --- | --- | --- | --- | --- |
|  |  | **Mean (SD)** | **Median (IQR)** | **Mean (SD)** | **Median (IQR)** |
| Variable costs |  |  |  |  |  |
| *Travel costs* |  |  |  |  |  |
| *Direct travel costs*** | $0.00 | $0.00 | $0.00 | $0.00 | $0.00 |
| *Time cost of travel* | $12,911.74 | $1844.53 ($410.72) | $1834.22 ($1649.96-$2030.65) | $4303.91 ($1142.56) | $4638.76 ($3835.05-$4940.20) |
| *Administrative time arranging travel*** | $0.00 | $0.00 | $0.00 | $0.00 | $0.00 |
| *In-person coaching* | $67,958.63 | $9708.38 ($1382.11) | $10168.58 ($8825.93-$10535.47) | $22652.88 ($4688.62) | $20772.32 ($19984.40-$24381.08) |
| *Preparatory work* | $12,582.98 | $1797.57 ($3143.36) | $1669.81 ($1404.38-$2142.40) | $4194.33 ($1326.77) | $3866.75 ($3464.39-$4760.47) |
| *Post-intervention evaluation* | $983.83 | $140.55 ($2893.96) | $121.18 ($106.06-$180.52) | $327.94 ($131.91) | $317.66 ($259.57-$391.18) |
| *Administrative time* | $55,246.91 | $7,892.42*** | $7,892.42*** | $18,415.64*** | $18,415.64*** |
| **Variable cost subtotal** | $149,684.09 | $21,383.44 ($2,025.75 | $22,003.69  ($20,157.97-$22,583.69) | $49,894.70 ($6,909.96 | $47,090.23 ($45,959.05-$52,428.11) |
| *Fixed costs* |  |  |  |  |  |
| *Curriculum development* | $5,587.70 |  |  |  |  |
| *Computer cost* | $1,524.12 |  |  |  |  |
| **Fixed cost subtotal** | $7,111.82 |  |  |  |  |
| **Total implementation cost** | **$156,795.91** |  |  |  |  |

Abbreviations: VAMC = Veterans Affairs Medical Center; SD = standard deviation; IQR = interquartile range

* Means and SD were not calculated as this was total aggregate cost, and not average

** Administrative time arrange travel and the cost of travel was assumed to be zero

*** Administrative time was divided across 7 VAMCs or 3 clusters as an estimate. No SD or IQR is reported.

**Sensitivity Analysis #2: Non-VA Setting Scenario**

Assumptions:

- National mean salaries used: $648,000 for interventional cardiologists, $72,080 for nurses/radiation technicians
- Conference room space used to deliver the coaching intervention was rented, at a rate of $50 per hour to rent federal facility conference rooms for less than 30 people.

***Variable, Fixed and Total Costs For the Full Study, per Site and per Randomization Cluster***

| **Cost Category** | **Full Study***  **(2019 dollars)** | **Per VAMC, n = 7 (2019 dollars)** | | **Per Cluster, n = 3 (2019 dollars)** | |
| --- | --- | --- | --- | --- | --- |
|  |  | **Mean (SD)** | **Median (IQR)** | **Mean (SD)** | **Median (IQR)** |
| Variable costs |  |  |  |  |  |
| *Travel costs* |  |  |  |  |  |
| *Direct travel costs* | $43,318.44 | $6188.35 ($1969.36) | $6806.67 ($4985.55-$7130.47) | $14439.48 ($3433.18) | $16042.69 ($13270.33-$16410.24) |
| *Time cost of travel* | $57,763.27 | $8251.90 ($1382.40) | $8473.39 ($7201.46-$9457.65) | $19254.42 ($5731.36) | $19440.77 ($16436.47-$22165.55) |
| *Administrative time arranging travel* | $124,022.46 | $17717.49** | $17717.49** | $41340.82** | $41340.82** |
| *In-person coaching* | $90,908.36 | $12986.91 ($992.58) | $13284.00 ($12519.51-$13485.07) | $30302.79 ($7614.85) | $27317.01 ($25975.15-$33137.54) |
| *Room rental* | $1,050.00 | $475.00 ($31.18) | $500.00 ($441.67-$500.00) | $350.00 ($0.00) | $350.00 ($350.00-$350.00) |
| *Preparatory work* | $16,392.83 | $2341.83 ($4590.12) | $2384.70 ($2213.54-$2741.84) | $5464.28 ($2056.62) | $4924.04 ($4327.87-$6330.57) |
| *Post-intervention evaluation* | $1,259.98 | $180.00 ($4380.42) | $175.54 ($175.54-$227.23) | $419.99 ($201.93) | $402.77 ($315.00-$516.38) |
| *Administrative time* | $50,085.99 | $7,155.14** | $7,155.14** | $16,695.33** | $16,695.33** |
| **Variable cost subtotal** | $384,801.34 | $55,296.62 ($3934.71) | $56757.14 ($53555.31-$57844.46) | $128267.11 ($17758.49) | $125145.54 ($118710.96-$136262.48) |
| *Fixed costs* |  |  |  |  |  |
| *Curriculum development* | $8,534.48 |  |  |  |  |
| *Computer cost* | $1,524.12 |  |  |  |  |
| **Fixed cost subtotal** | $10,058.60 |  |  |  |  |
| **Total implementation cost** | **$394,859.94** |  |  |  |  |

Abbreviations: VAMC = Veterans Affairs Medical Center; SD = standard deviation; IQR = interquartile range

* Means and SD were not calculated as this was total aggregate cost, and not average

** Administrative time was divided across 7 VAMCs or 3 clusters as an estimate. No SD or IQR is reported.

**Sensitivity Analysis #3: Training All Cardiologists Scenario**

Assumptions:

- At each site, there were 4.7 interventional cardiologists (mean number at each study site) that participated in the intervention.
- The salary of cardiologists at a given site was the mean salary at that site.

***Variable, Fixed and Total Costs For the Full Study, per Site and per Randomization Cluster***

| **Cost Category** | **Full Study***  **(2019 dollars)** | **Per VAMC, n = 7 (2019 dollars)** | | **Per Cluster, n = 3 (2019 dollars)** | |
| --- | --- | --- | --- | --- | --- |
|  |  | **Mean (SD)** | **Median (IQR)** | **Mean (SD)** | **Median (IQR)** |
| Variable costs |  |  |  |  |  |
| *Travel costs* |  |  |  |  |  |
| *Direct travel costs* | $71,507.01 | $10215.29 ($3041.21) | $9447.37 ($8187.54-$11874.80) | $23835.67 ($5189.73) | $25822.45 ($21884.26-$26780.47) |
| *Time cost of travel* | $86,062.39 | $12294.63 ($2380.57) | $12405.17 ($10475.30-$13836.65) | $28687.46 ($5089.58) | $26070.65 ($25754.66-$30311.86) |
| *Administrative time arranging travel* | $143,404.69 | $20486.38** | $20486.38** | $47801.56** | $47801.56** |
| *In-person coaching* | $165,520.16 | $23645.74 ($3035.00) | $23793.03 ($21738.66-$26046.19) | $55173.39 ($11515.53) | $50661.41 ($48629.23-$59461.55) |
| *Preparatory work* | $44,806.25 | $6400.89 ($8094.01) | $6614.59 ($5768.13-$6981.51) | $14935.42 ($3517.47) | $12916.48 ($12904.62-$15956.75) |
| *Post-intervention evaluation* | $4,224.06 | $603.44 ($7679.19) | $620.46 ($539.67-$659.61) | $1408.02 ($345.51) | $1227.66 ($1208.84-$1517.03) |
| *Administrative time* | $55,246.91 | $7,892.42** | $7,892.42** | $18,415.64** | $18,415.64** |
| **Variable cost subtotal** | $570,771.48 | $81538.78 ($8131.72) | $84860.77 ($74642.79-$87786.57) | $190257.16 ($23150.21) | $184770.50 ($177556.83-$200214.16) |
| *Fixed costs* |  |  |  |  |  |
| *Curriculum development* | $5,587.70 |  |  |  |  |
| *Computer cost* | $1,524.12 |  |  |  |  |
| **Fixed cost subtotal** | $7,111.82 |  |  |  |  |
| **Total implementation cost** | **$577,883.29** |  |  |  |  |

Abbreviations: VAMC = Veterans Affairs Medical Center; SD = standard deviation; IQR = interquartile range

* Means and SD were not calculated as this was total aggregate cost, and not average

** Administrative time was divided across 7 VAMCs or 3 clusters as an estimate. No SD or IQR is reported.

**Sample Agenda 1: In-person Team-Based Training**

Implementation Trial of a Coaching Intervention to Increase the Use of Transradial PCI

7:30am Registration, meet & greet

8am Welcome & Introduction

8:05am Suicide Prevention

8:20am Making the Case for TRI

8:45am Patient Selection & Prep

9:05am Access & Closure

9:25am TRI- Diagnostic Angiography

9:45am TR-PCI and RHC

10am Break

10:15am Live Cases & Simulator Session

12:15pm Lunch Break

12:45pm TR-STEMI

1:15pm TR Complications

1:35pm Discharge Process

2pm Same Day Discharge Program

2:30pm Live Cases & Simulator Session

4pm Course is complete

Voluntary Q&A session with Clinical Coaches & Principal Investigators

**Sample Agenda 2: Coaching Site Visit**

Implementation Trial of a Coaching Intervention to Increase the Use of Transradial PCI

*This is a draft agenda & can be adjusted as needed to meet the needs of the team. We would like to be present for two patients however they are scheduled.*

| Time | Agenda item | Notes |
| --- | --- | --- |
| •7:15 – 8:00 | Review goals of transradial approach | Go over agenda for the day; equipment list; how it has gone since team-based training? |
| •8:00 – 10:00 | Patient 1 | Any information about the case to review ahead of time? |
| •10:00 – 11:00 | Debrief: Policy, Procedures, Protocols | Review the fidelity checklist |
| •11:00 – 12:00 | Debrief: Pre-and Post Procedure Care | Patient selection, pre-procedure education, what to expect during the procedure, and post-procedure care. Discharge instructions to patients. |
| •12:00 – 1:00 | Lunch Q and A |  |
| •1:00 – 2:30 | Patient 2 | Any information about the case to review ahead of time? |
| •2:30 – 3:00 | Wrap up, next steps, facilitators and barriers | Review how to gradually increase the complexity of the patients TRA performed on. |
